# Recent Advances in the Design of Plasmonic Au/TiO_2_ Nanostructures for Enhanced Photocatalytic Water Splitting

**DOI:** 10.3390/nano10112260

**Published:** 2020-11-15

**Authors:** Jehad Abed, Nitul S Rajput, Amine El Moutaouakil, Mustapha Jouiad

**Affiliations:** 1 Department of Materials Science and Engineering, University of Toronto, Toronto, ON M5S 3E4, Canada; jehad.abed@mail.utoronto.ca; 2 Department of Mechanical Engineering, Masdar Institute part of Khalifa University of Science and Technology, Abu Dhabi 54224, UAE; nitul.rajput@ku.ac.ae; 3 Department of Electrical Engineering, UAE University, Al Ain 15551, UAE; a.elmoutaouakil@uaeu.ac.ae; 4 Laboratory of Physics of Condensed Mater, University of Picardie Jules Verne, 80039 Amiens, France

**Keywords:** water splitting, photocatalyst, plasmonics, Au/TiO_2_ nanostructures, Back silicon, photocurrent, hot electrons

## Abstract

Plasmonic nanostructures have played a key role in extending the activity of photocatalysts to the visible light spectrum, preventing the electron–hole combination and providing with hot electrons to the photocatalysts, a crucial step towards efficient broadband photocatalysis. One plasmonic photocatalyst, Au/TiO_2_, is of a particular interest because it combines chemical stability, suitable electronic structure, and photoactivity for a wide range of catalytic reactions such as water splitting. In this review, we describe key mechanisms involving plasmonics to enhance photocatalytic properties leading to efficient water splitting such as production and transport of hot electrons through advanced analytical techniques used to probe the photoactivity of plasmonics in engineered Au/TiO_2_ devices. This work also discusses the emerging strategies to better design plasmonic photocatalysts and understand the underlying mechanisms behind the enhanced photoactivity of plasmon-assisted catalysts.

## 1. Introduction

Water splitting (WS) powered by solar irradiation has emerged as a clean route towards a green hydrogen economy [1]. Since Fujishima and Honda first demonstrated water splitting using TiO_2_ [2], several studies have used TiO_2_ and other semiconductors such as ZnO to achieve efficient WS. TiO_2_ is one of the most investigated photocatalyst systems due to its superior electronic and chemical properties. However, the solar collection efficiency of TiO_2_ is limited to UV spectrum due to its wide band gap 3.2 eV covering only 4–5% of the total solar spectrum. Extending the photoactivity of TiO_2_ beyond Ultraviolet (UV) can be achieved by several strategies, such as using electron donor/sacrificial agents to assist in the activity of the WS [3,4,5,6,7], dye sensitization [8,9,10], Nobel metal loading [11,12], doping [13], and stacking of heterojunction semiconductors [14]. 

Enhancing solar light harvesting by decorating catalysts with plasmonic nanostructures such as Au and Ag nanoparticles has been extensively studied in the last decades. Au and Ag, in addition to other transition metals such as Cu, not only can work as hole traps to prevent recombination but also exhibit interesting plasmonic properties that can extend the photoactivity of wide-bandgap semiconductors from the UV light to IR, accounting for more than 45% of the solar spectrum. Surface plasmons excited at the interface of these metal/semiconductor nanostructures can decay into hot electrons that have gained a very high kinetic energy and accelerated by intense electric fields at the surface of the plasmon, with energies higher than the Schottky barrier of the metal/semiconductor interface [15,16,17]. Several plasmonic nanostructures were reported to enhance the photocatalytic activity of hydrogen evolution reaction (HER) catalysts [18]. For instance, in the study carried out by Ingram et al. [19], Ag plasmonic nanoparticles were used in conjunction with TiO_2_ to enhance the HER. Similarly, hierarchical TiO_2_ nano-architecture loaded with Pt nanoparticles were observed to dramatically enhance the hydrogen production [20]. In the case of TiO_2_ nanostructures loaded with Au, the plasmonic activity of Au nanoparticles and their shape and size were attributed to the significant increase in hydrogen production [21,22,23,24]. These studies clearly indicate the importance of the role of plasmonic of nanoparticles, in particular in Au/TiO_2_ systems, on enhancing photocatalytic activity of HER catalysts.

Investigating the plasmonic behavior of the metallic nanostructures is of great importance as it provides with feedbacks and inputs allowing to design efficient devices based on MDPhC. The MDPhC and nanostructure configurations considered in this review are based on TiO_2_ acting as the WS catalyst and Au nanostructure as plasmonic material. In the following, the principle of plasmonics behavior of Au nanoparticles is detailed, as well as their interaction with TiO_2_ as photocatalyst to achieve WS process. Briefly, the principle of WS is tackled especially in the presence of plasmonics, and examples of MDPhC designs consisting of TiO_2_ catalyst and Au are given and discussed in the light of the role of LSPR in achieving efficient WS.

## 2. Plasmonics and Water Splitting 

Many emerging areas of nanotechnology are focused on utilizing and understanding plasmonic properties for optical and catalytic applications [25,26,27]. Generally, surface plasmons are generated by external excitations (photons or electrons) of the conduction band electrons at the surface of the metal. Propagating plasmons on the surface of a metal are called surface plasmon polaritons (SPPs). Their oscillations are associated with a large enhanced electric field that decays exponentially in the perpendicular direction of the metal/semiconductor interface. Hence, they are very sensitive to the changes in the environment near the interface with the dielectric. Plasmons can resonate with incident light producing an amplified oscillation of electrons in the conducting band by surface plasmon resonance (SPR); this resonance can be localized at the surface of the metal (non-propagating waves) if the dimension of the metal particles is smaller than the wavelength length of the incident light resulting in localized surface plasmon resonance (LSPR). The resonance frequency of LSPR depends on the particles’ size, shape, and defects and the surrounding environment [19,28,29]. Several attempts have been made to synthesize efficient plasmonic metal-semiconductor systems, usually nanostructures made of noble metals such as Au, Ag, and Pt [15,19,20,30,31,32,33,34,35,36,37,38]. These metals exhibit plasmons resonance in the visible region while also possessing very good physical/chemical properties such as corrosion and oxidation resistance for Au, intense LSPR for Ag, and enhanced catalytic properties for Pt. More elements with large negative real dielectric constant and a small imaginary component have been shown to possess plasmonic properties such as Rh, Pd, Al, and Cu. [30,39,40,41,42,43,44]. However, these metals are not stable and suffer from severe corrosion and oxidation in contact with water during WS experiments, especially Al and Cu, making them less attractive for the photocatalytic WS application in comparison to Au/TiO_2_ [45].

Metal/semiconductor nanocomposites have demonstrated great strides in improving the photoactivity and charge separation of catalysts for efficient WS [46,47,48,49]. The electrochemical dissociation of water to evolve H_2_ and O_2_ requires a thermodynamic potential of 1.23 V between two electrodes, a cathode electrode for the hydrogen evolution reaction (HER), and an anode electrode for oxygen evolution reaction (OER). The mechanism of water splitting on TiO_2_ is shown in the following chemical reactions:

UV excitation of TiO_2_:

2TiO_2_ + 4h → 4e^−^ + 4p^+^ (1)

O_2_ evolution at the anode:

4p^+^ + 2H_2_O → O_2_ + 4H^+^ (2)

H_2_ evolution at the cathode:

4e + 4H^+^ → 2H_2_ (3)

Overall reaction:

2H_2_O → 2H_2_ + O_2_ (4)

**Figure 1:**
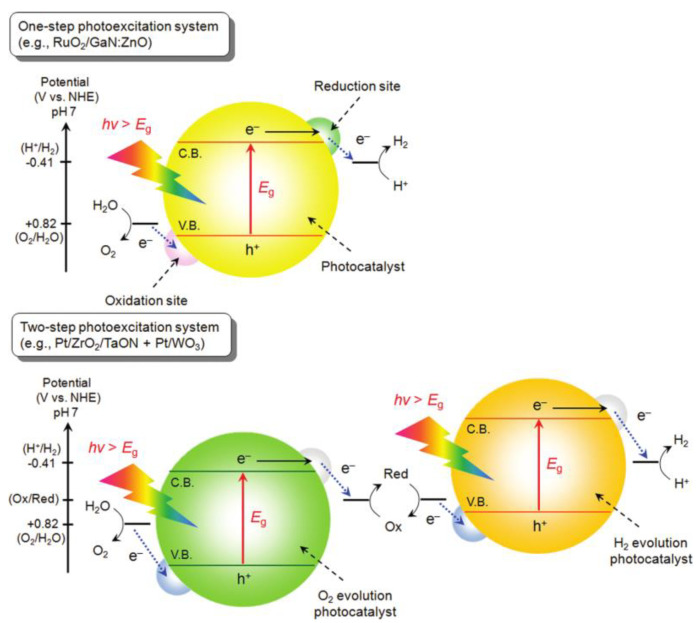
Schematic diagram of one- and two-step photocatalytic water splitting. Reproduced from [51]. with permission from American Chemical Society, 2010.

Semiconductors with a suitable band gap (>1.23 eV) such as TiO_2_, ZnO, and CdS can act as potential photocatalysts and split water molecules using UV light [50]. In addition to the band gap requirement, the unique electronic properties of semiconductors only allow one- or two-step photoexcitation on the surface of the photocatalyst, as shown in Figure 1. 

Unlike two-step photoexcitation, one-step photoexcitation creates anodic/oxidation and cathodic/reduction sites on the surface of the photocatalyst to split water. When light is irradiated on the surface of the semiconductor, electrons are excited from the valence band to the conduction band leaving behind positive holes. To satisfy the one-step photocatalytic WS condition, the top-edge of the valence band (anodic site) should be positively charged above the water oxidization potential (EO_2_/H_2_O = 1.23 V vs. reversible hydrogen electrode at pH = 0) and the bottom-edge of the conduction band (cathodic site) should be more negative than the reduction potential of H+ to H_2_ (EH+/H_2_ = 0 V vs. reversible hydrogen electrode at pH = 0). In addition to the electronic properties of the semiconductor, the overall efficiency of WS is highly dependent on the physical and chemical properties of the semiconductor such as photo-corrosivity, chemical and thermal stability, electron/hole lifetime, etc. [7]. As expected from this proposed scheme, many photocatalysts of interest such as TiO_2_ are limited to the UV portion of the solar spectrum, which only covers ca. 4% of the total solar spectrum. In the last decade, several mechanisms and various materials were proposed to develop photocatalysts capable of splitting water under visible light irradiation, which accounts for more than 45% of the solar spectrum [52,53,54,55,56,57,58]. 

**Figure 2:**
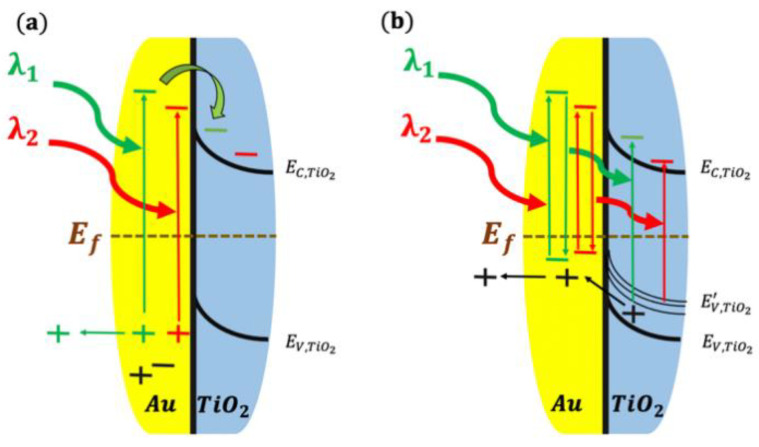
Au/TiO_2_ interface band diagram: (**a**) direct electron transfer; and (**b**) plasmonic resonance electron transfer. Adapted from [60]. with permission from Springer Nature, 2016.

Noble metal nanoparticles such as Au and Ag are usually used to extend the photoactivity of TiO_2_ to the visible light since they possess LSPR under visible light irradiation. At first, the nanoparticles absorb the light through the plasmonic resonance process. These resonance modes (periodic oscillation of the electron cloud) at the surface of the nanoparticles exhibit a strong exponential decay within the metal/semiconductor interface (~10 nm in the semiconductor direction) [59]. Subsequently, in the second phase, the plasmon decays with time by transferring the amassed energy to the electrons in the conduction band of the metal nanoparticles. The strong field localization and enhancement around metallic nanoparticles excites electrons from the fermi level of the metal to higher energy in the conduction band within few nanometers in the semiconductor, as shown in Figure 2.

Injected (transferred) electrons are strongly excited with high kinetic energies and so referred to as “hot” electrons. In the third phase, the hot electrons, which are relatively more energetic than the free electrons, can jump to the semiconductor by overcoming the Schottky barrier. Thus, these hot electrons play an important role in achieving charge separation and enhanced photocurrents by overcoming the Schottky barrier at the metal/semiconductor junction. For example, in the case of the Au/TiO_2_ plasmonic WS system, the Schottky barrier is around ~1.1 eV. LSPR of Au under visible light irradiance can produce hot electrons with energies of 1–3.5 eV, which is sufficient to overcome the barrier. This electron injection process achieved from the metal to the semiconductor can occur through two mechanisms: direct electron transfer (DET) and plasmonic resonance electron transfer (PRET) (Figure 2a,b respectively). In the former process, electrons are directly and physically transferred to the conduction band of the semiconductor through the interface. This process allows the semiconductor (TiO_2_) to have active electrons and continue the WS process. On the other hand, in PRET, the electrons do not transfer physically to the semiconductor; instead, the energy of the hot electrons is transferred to the electrons in the valence band of the semiconductor and becomes excited to the conduction band of the semiconductor. PRET stimulates interband transition within the semiconductor to the conduction band and does not require direct contact between the metal and the semiconductor. Moreover, the absorption spectra of the semiconductor TiO_2_ and Au have to overlap to facilitate PRET [60,61,62]. It is worth noting that both DET and PRET are competitively participating to hot electron injection making the deconvolution of their respective contribution to hot electrons injection very challenging. Nevertheless, DET and PRET have different activation requirements. For instance, in DET mechanism, the Au nanostructures have to be in physical contact with TiO_2_, hence a lack of contact will slow DET mechanism. Furthermore, it was reported that, for Au/TiO_2_, the hot electrons injected into TiO_2_ through DET mechanism have longer lifetime as compared to electrons photogenerated by the intraband transition in TiO_2_ [63]. In contrast, for PRET mechanism, hot spots generated in the metallic particles (Au) extends to TiO_2_ [60], which means that no direct contact between the metal and the semiconductor is essential. Hence, PRET can proceed despite the presence of a thin insulating layer between the plasmonic metal and the semiconductor [64]. The thin insulating layer was reported to be as thick as ~25 nm [65]. Additionally, PRET mechanism is always active as long as there is a spectral overlap between the LSPR and the absorption band of the semiconductor. To summarize, the contribution from PRET highly depends on two main factors: the physical separation between the metal and the semiconductor and the absorption spectral overlap between LSPR and the semiconductor. In the case of Au/TiO_2_, due to the large band gap of TiO_2_ (~3.2 eV), resonant energy transfer to TiO_2_ through the absorption of visible light through Au has a low probability of happening. However, it is expected that, if multiple plasmons decay at the same time, then all the energies can be combined to excite an electron hole pair in the semiconductor (TiO_2_). Hence, depending on the configuration of the Au/TiO_2_ system, both DET and PRET can actively participate in the photocatalytic activity. In a study by Cushing et al. [66], a design chart was established to accurately predict the presence of each mechanism. In their work, high degree of PRET activity was demonstrated in metal@TiO_2_ core–shell particles. The real mechanism of hot electrons injection from Au to TiO_2_ remains an area of great controversy and is still highly debated.

## 3. Design of Plasmonics Based Water Splitting Devices

For a practical water splitting device to operate efficiently, a proper design has to be implemented. In general, material modification along with morphological design can effectively increase the photocatalytic reaction of WS. Photon absorption capability, which is essential for enhancing the photoactivity during WS reaction, can be achieved by material engineering of the TiO_2_-Au systems. Particularly, the plasmonic activity of metals depends on the shape and size of the nanostructures [67,68,69,70]. Thus, it is crucial to practice judicial control over the design of Au/TiO_2_ to optimize the photoactivity. Moreover, extending the absorption spectrum of photocatalysts can be controlled by tailoring the morphology and crystal structure of the catalyst (TiO_2_) as well as the shape and size of the plasmonic particles (Au). Thus, a device needs to be designed that can absorb the light at full solar spectrum range. When light is impinged on the surface of the photocatalyst, a portion of the light is either reflected or scattered reducing the number of absorbed photons for the water splitting reaction, hence energy is lost. By controlling the shape of the photocatalyst, reflection and scattering can be suppressed to enable a larger portion of the photons be absorbed for water splitting. For instance, a vertically standing cup-like structure or a structure with zigzag morphology could boost absorption by light trapping. Below, we discuss the effects of nanoengineering the morphology, crystal structure, and particle shape on the overall performance of photocatalytic water splitting.

### 3.1. TiO_2_-Au System

**Figure 3:**
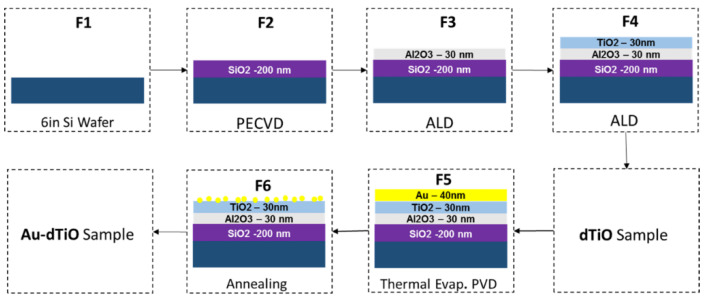
Fabrication steps to produce dTiO sample and Au-dTiO sample: F1, a 6-inch Si water; F2, 200 nm of SiO_2_ was deposited on Si; F3, 30 nm alumina was deposited on SiO_2_/Si; F4, TiO_2_ deposited sample (dTiO); F5, 40-nm Au deposited on dTiO; F6, annealed sample (Au-dTiO). Reproduced from [71] with permission from Elsevier, 2018.

To study the effect of crystal structure on the overall WS activity of Au/TiO_2_ photocatalysts while excluding morphology effects, a stacked device of Au nanoparticles on top of atomically flat TiO_2_/Al_2_O_3_/SiO_2_/Si was fabricated. The fabrication steps are shown in Figure 3.

**Figure 4:**
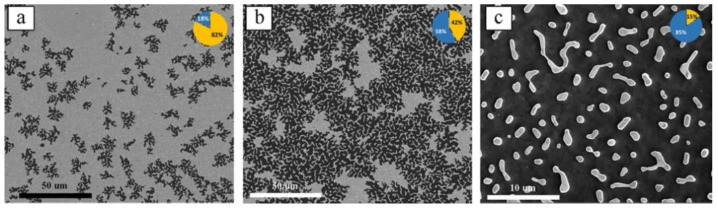
(**a**) SEM top view image of Au annealed for 2 h at 450 °C (the inset shows surface area ratio of Au (yellow) to TiO_2_ (blue)); and (**c**) Au-dTiO annealed at 450 °C for 6 h. Reproduced from [71] with permission from Elsevier, 2018.

In the first step, the Plasma Enhanced Chemical Vapor Deposition (PECVD) process was used to deposit a 200-nm SiO_2_ layer on Si wafer. Then, Atomic Layer Deposition (ALD) was used to deposit Al_2_O_3_ and TiO_2_ to obtain the dTiO sample (dTiO: TiO_2_/Al_2_O_3_/SiO_2_/Si). Next, Au was thermally evaporated on the stack. Then, the samples were annealed at different times and temperatures to control the crystal structure and the morphology of the grown material (Au-dTiO). Due to annealing, the Au thin film shrunk to form islands and particles, as shown in Figure 4. As the annealing temperature was increased, more random dendrite-like Au nanostructures evolved with various sizes and orientations, which is desired to achieve a broadband absorption of light as LSPR resonance frequency strongly depends on the particles size and shape [72,73,74,75,76]. The LSPR contribution from individual Au nanostructure sums up collectively to enhanced absorption over a broad span of the solar spectrum. In summary, an optimization of the sample annealing was required to keep larger effective surface area of TiO_2_ for increased interaction with water while maintaining enough Au particles for plasmonic contribution. Eventually these plasmonic particles actively participate in enhancing the WS activity.

**Figure 5:**
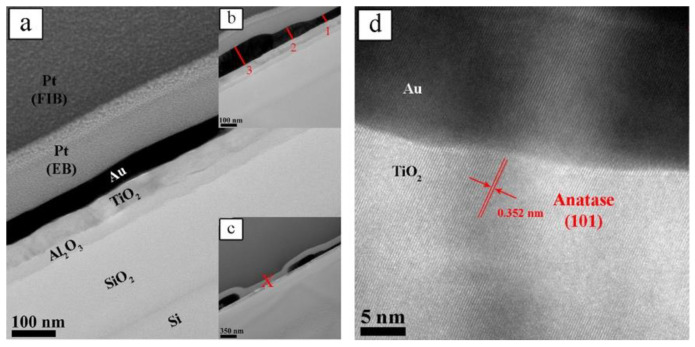
TEM cross sectional images of Au-dTiO annealed sample (annealed at 450 °C for 6 h): (**a**) continuous and uniform gold film; (**b**) thickness variation at specific locations; (**c**) discontinuity in the gold film; and (**d**) HRTEM image of the TiO_2_/Au interface. Reproduced from [71]. with permission from Elsevier, 2018.

Cross-sectional view of an annealed sample is shown in Figure 5a–c. The thicknesses of the layers can be determined from the images. However, it is also found that the thickness of the Au layer varies spatially. In Figure 5d, one can notice that the annealing had induced the coarsening of Au particles by Oswald ripening mechanism as well as the crystallization in the TiO_2_ layer. Prior studies reported that crystalline TiO_2_ in anatase and/or rutile form exhibits superior optical properties compared to its amorphous counterpart [77,78].

**Figure 6:**
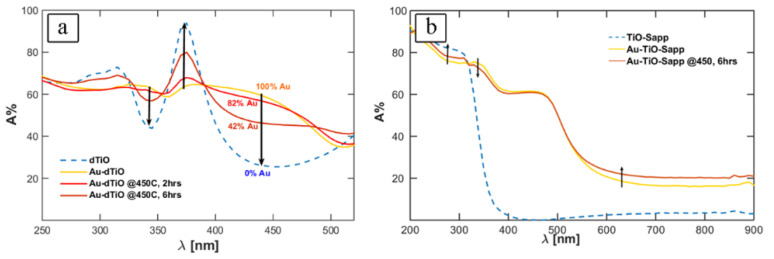
(**a**) Optical absorption spectrum of dTiO and Au-dTiO samples; and (**b**) optical absorption spectrum of TiO-Sapp and Au-TiO-Sapp samples. Reproduced from [71]. with permission from Elsevier, 2018.

The overall performance of the as-fabricated and -modified Au/TiO_2_ composite was assessed through various techniques. Optical absorption spectroscopy and quantum efficiency measurements were used to evaluate the performance. Controlling Au:TiO_2_ surface area coverage by annealing is important to optimize both optical absorption and the exposure of TiO_2_ sites for water splitting. The highest broadband absorption occurs when a thin layer of Au is covering TiO_2_ completely (100% Au), as shown in Figure 6a. After annealing, more of TiO_2_ surface was exposed to light resulting in the emergence of an absorption peak at around 375 nm while absorption dipped within 345–440 nm due to a shrunken Au surface coverage. It was found that annealing at 450 °C for 6 h provided a balance between absorption and exposed TiO_2_ sites. To eliminate interferometric behavior between different absorbing layers in the stack, Au/TiO_2_ were deposited similarly to the stack but on a sapphire substrate for absorption spectroscopy investigations, as shown in Figure 6b. The absorption behavior of annealed samples on sapphire showed that the formation of Au nanoislands with various sizes and shapes extended the optical absorption spectrum of light in the visible region.

**Figure 7:**
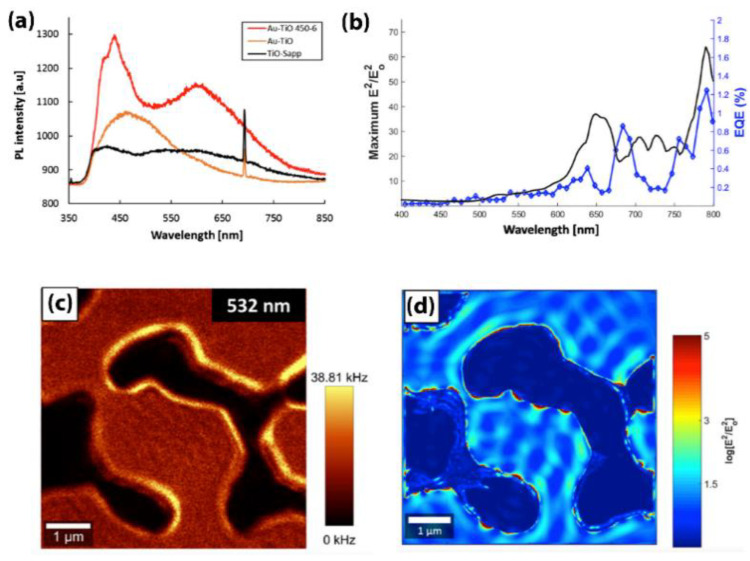
Annealed Au/TiO_2_ responses: (**a**) photoluminescence; (**b**) external quantum efficiency (EQE); (**c**) A SNOM map; and (**d**) corresponding FDTD simulation. Reproduced from [79]. with permission from Springer Nature, 2018.

To understand the nature of the excited plasmon modes and their dependence on the size and the shape of Au nanostructures, scanning near-field optical microscopy (SNOM), photoluminescence, and quantum efficiency tests were conducted in the study and validated by finite domain time difference (FDTD) simulations, as shown in Figure 7.

Photoluminescence activity of three different samples were measured: TiO_2_ deposited on sapphire sample (TiO-Sapp), Au deposited on TiO_2_/Sapphire (Au/TiO), and annealed Au/TiO sample at 450 °C for 6 h. All the samples were photoexcited by a 355-nm laser. They exhibited an onset value of 380 nm corresponding to the bandgap of TiO_2_ (3.2 eV). The as-deposited Au/TiO sample gave a peak at around 460 nm, which corresponds to the interband transitions between d-bands and sp conduction bands [80]. Two strong peaks appeared for the annealed Au/TiO sample. The first peak (450 nm) is similar to the interband transitions. The second peak appeared at 630 nm and can be attributed to the surface plasmonic activity of Au [80]. Then, external quantum efficiency (EQE) measurement was conducted on the annealed Au/TiO sample (Figure 7b). The measurement was carried out in dry environment using Xenon light monochromatic source. Both EQE and FDTD simulations showed an increase in the photoactivity of annealed Au/TiO after 570 nm in the form of multipeaks. Each peak could be attributed to the plasmonic activity of Au nanostructures with different sizes and shapes. Using SNOM, a hot spot absorption map was constructed for an Au nanostructure, as shown in Figure 7C. The SNOM map and FDTD confirmed the presence of surface plasmons in the nanostructure when excited with an external light source. This study used SNOM, FDTD, and EQE to investigate the plasmonic contribution of Au nanoparticles on enhancing the photocatalytic activity of TiO_2_.

### 3.2. Black Silicon Combined with Au/TiO_2_

**Figure 8:**
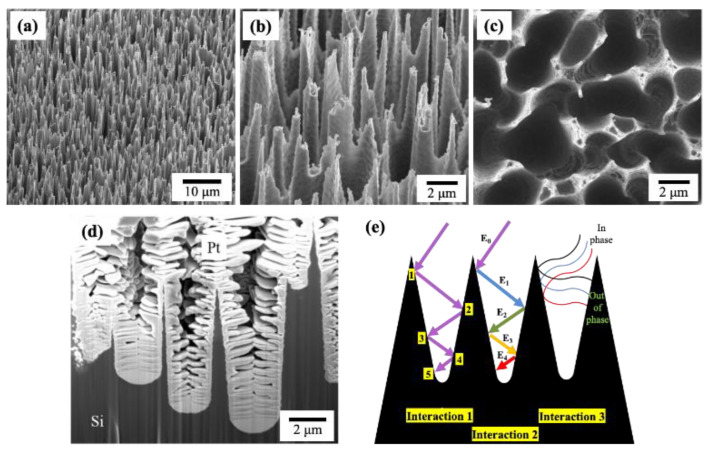
(**a**,**b**) SEM images (tilted view) of a BSi sample showing the morphology obtained after a selective etching of Si; (**c**) a top view image of the sample; (**d**) a cross-sectional view shows the sharp Si peaks and deep wells formed after the etching process; and (**e**) schematic picture showing light absorption through three interaction types. Adapted from [82] with permission from Elsevier, 2018.

In this study, a strategy to control the morphology of the substrate (Si) to improve light interaction and absorption was considered. Black Silicon (BSi) is a deeply etched Si surface, fabricated from N-type silicon by Deep Reactive Ion Etching (DRIE) technique using medium density plasma, with fluorine as an etchant [81]. An SEM image of a BSi is shown in Figure 8. Figure 8a–c shows, respectively, tilted views and top view of BSi surface. The etching resulted in the formation of sharp spikes with deep cavities, as illustrated in the cross-sectional image of Figure 8d. The height of Si spikes can be as long as few microns, where the deep wells act as a “trapping potential” to prevent the photons from escaping the material. When an impinging photon enters inside wells, it starts interacting with the surface of BSi; as a result, various types of light–matter interactions may occur (Figure 8e). 

**Figure 9:**
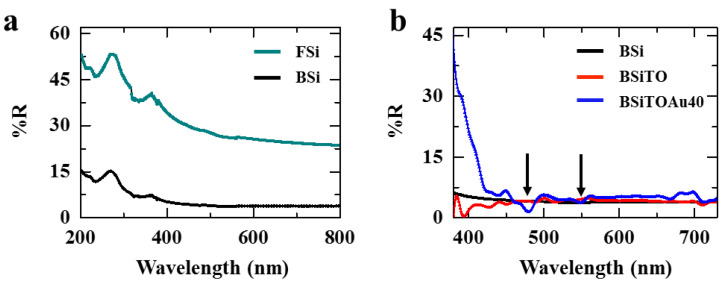
Comparison of optical spectra obtained from: (**a**) FSi and BSi; and (**b**) BSi, BSiTiO_2_ (BSiTO), and BSiTiO_2_-Au 40 nm (BSiTOAu40) samples. The arrow marks indicate the enhancement of absorption in the BSiTOAu40 sample after adding the Au nanoparticles. Adapted from [82] with permission from Elsevier, 2018.

First, multiple interactions between a single photon and the material may take place leading to an increase of the probability of the absorption (Interaction 1). Then, an energetic photon can lose its energy while sinking into the well (Interaction 2). This photon does not have sufficient energy to escape the well, hence it is trapped inside the BSi. Moreover, because of the gradual change of refractive index along the length of BSi, the waves become out of phase and cause destructive interference, thus lowering the energy of the waves and provoking an additional trapping of the photons (Interaction 3). Therefore, BSi is a smart photonic design for increasing light absorption. Optical spectra comparing the light absorption on flat silicon (FSi) and BSi are shown in Figure 9a. One can notice from this figure that there is a dramatic increase in light absorption for BSi achieved by surface engineering.

Following the fabrication of the etched Si substrate, the photocatalyst TiO_2_ was deposited. A thin film of 40 nm was grown using ALD process. Then, Au was deposited on top of the TiO_2_ film. The thickness of the Au was kept within 40 nm which provides with a good compromise between the light absorption intensity and electrical conductivity of the photocatalyst. To assess the performances of the device, absorption spectroscopy and photocurrent measurement were carried out. Figure 9b shows that the addition of 40 nm of Au improved the absorption in the visible range at around 480 and 550 nm (marked by the arrows in the Figure 9b).

**Figure 10:**
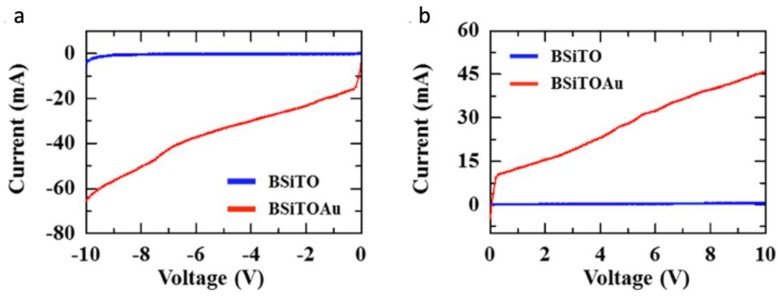
Photocurrents measured in both negative (**a**) and positive (**b**) voltage on BSi loaded with 40 nm of TiO_2_ and 40 nm of Au. Adapted from [82] with permission from Elsevier, 2018

To further confirm the efficient light absorption proprieties of BSi, a photocurrent measurement was carried out to evaluate the device generated current by photoexcitation. A Keithley source meter, consisting of a four-probe measurement setup, was utilized to capture the photo-induced current. In this experiment, the current was monitored with light off (using dark room) and light on using a broad band light source (Xenon lamp). To underline the contribution of the plasmonic Au particles, two samples were considered: a clean BSi sample coated with TiO_2_ (BSiTO) and a coated BSiTO sample loaded with Au nanoparticles (BSiTOAu). The deposited Au material was monitored to obtain nanostructures with various shapes and sizes in order to enhance the light absorption through plasmonic effect. As one can notice in Figure 10, the resulted photocurrent greatly increased for BSiTOAu sample, indicating a significant effect of Au nanostructures. Hereby, these results demonstrate on the one hand that BSi compared to FSi substrate is far superior in terms of light absorption rendering it a good candidate for efficient photocatalyst and on the other hand that TiO_2_ coated BSi and loaded with Au nanostructures exhibited high photo-induced current. This also indicates that BSi is a potential substrate/carrier to host Au/TiO_2_ WS systems as it considerably enhances light absorption and subsequently increases the photocurrent activity in the device [82].

### 3.3. Design of Cavity Shaped Au/TiO_2_ Device

**Figure 11:**
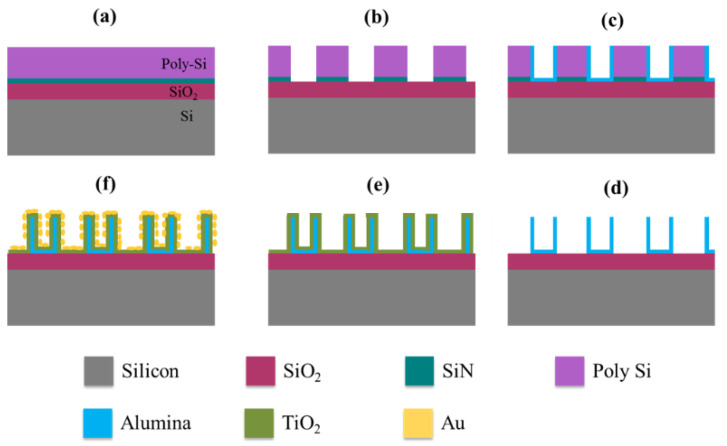
Schematic pictures of the fabrication steps: (**a**) ~100 nm of thermally deposited SiO_2_, ~150 nm of SiN, and 500 nm of poly Si were deposited via low pressure chemical vapor deposition (LPCVD) process. (**b**) The poly Si layer was subsequently patterned with a UV stepper and then etched with RIE. The SiN layer was etched due to over etching. (**c**) Nearly 40 nm of Al_2_O_3_ (alumina) was conformally deposited using atomic layer deposition (ALD) and then anisotropically etched using RIE, leaving only the sidewalls. (**d**) The poly Si layer was etched via XeF_2_ gas etching leaving only vertically the standing alumina sidewalls. (**e**) Then, TiO_2_ was deposited on the alumina shells using the isotropic ALD deposition. (**f**) Finally, the structures were decorated with Au nanoparticles by using a sputter deposition.

Parallel to the promising results obtained on BSi sample, a novel design of platform to host Au/TiO_2_ system with cavity-like shape was engineered to amplify the sunlight trapping. In this sense, alumina shells were prepared using a sidewall lithography process [83,84]. These shells were utilized as supporting figures to host the TiO_2_-Au system. A series of cleaning, depositing, and etching steps was carried out to obtain the vertically standing alumina shells. At first, a Si layer was deposited on the top of Si/SiO_2_ substrate. Then, low-pressure chemical vapor deposition (LPCVD) was employed to grow the Si layer. Subsequently, a photomask was applied to generate circular pattern to evenly etch the exposed Si region. Thereafter, an alumina layer was deposited uniformly across the surface. The next step consisted of an anisotropic etching to remove the remaining Si, leaving the vertically standing thin alumina shells. Once the alumina shells were fabricated, a thin TiO_2_ layer was deposited using ALD process, followed by annealing. The final stage of fabrication, a deposition of thin Au layer (nanoparticles), was realized by means of magnetron sputtering. The step-by-step process is shown in the schematic diagram of Figure 11.

**Figure 12:**
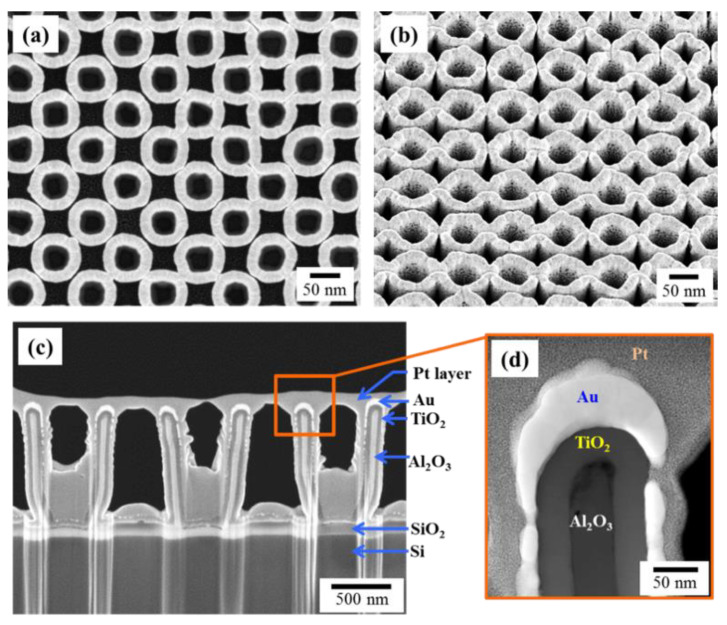
(**a**) A top view SEM image of the fabricated cup-like water splitting nanostructures. (**b**) Tilted SEM image of the sample. (**c**) Cross-sectional view of a thin slice of the nanostructures prepared using FIB deposition and milling, showing the structure and different layers. (**d**) High magnification STEM image taken from the region indicated by the orange box in (**c**). Adapted from [86] with permission from the PCCP Owner Societies, 2017.

The SEM images of the fabricated nanostructures are given in Figure 12a–c. The multi-layered structure is visible through the cross-sectional view shown in Figure 12c. In the high-resolution STEM, the deposited Au region appears to resemble an inverted fishing hook (FH). The images were collected from the TEM lamella sample, prepared using a dual focus ion beam (FIB) system [85]. 

**Figure 13:**
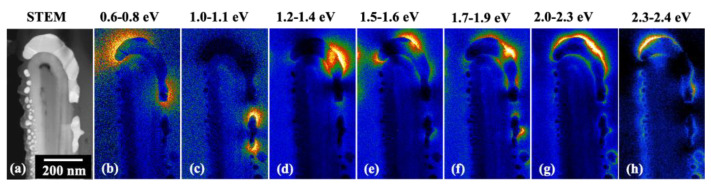
(**a**) STEM image shows the inverted FH structure and the presence of other Au nanoparticles. (**b**–**h**) Plasmonic modes observed at different energy widows. Each EF map is represented in temperature mode, the color scales are independent of each other, and higher electron energy loss events are shown in yellow, while the dark blue regions indicate lower energy loss events. Adapted from [86] with permission from the PCCP Owner Societies, 2017.

Subsequently, HRTEM and X-ray diffraction analyses revealed that the as-deposited TiO_2_ layer appeared to be amorphous and the Au nanostructures are polycrystalline. The sputtered Au nanoparticles displayed different sizes and shapes and mostly in particulate form. To evaluate the Au plasmonic contribution, electron energy loss (EELS) investigations were carried out. EELS technique allows precise mapping of the plasmonic hot spots in the WS Au/TiO_2_ based systems. To demonstrate the presence of plasmonic effects in the as-grown cup-like nanostructures, double-corrected HRSTEM system operating at 80 kV was used to trigger the plasmonic hot spots using high energetic electron beam excitation. The Energy Filtered (EF) plasmon maps for the FH-like shape and the asymmetric particle (AS) present inside the nanostructure cavities are given in Figure 13b–h. The FH-like structure presented an active source for plasmonic hotspots at various energy windows, whereas the AS particle provided hot spots for only two modes.

**Figure 14:**
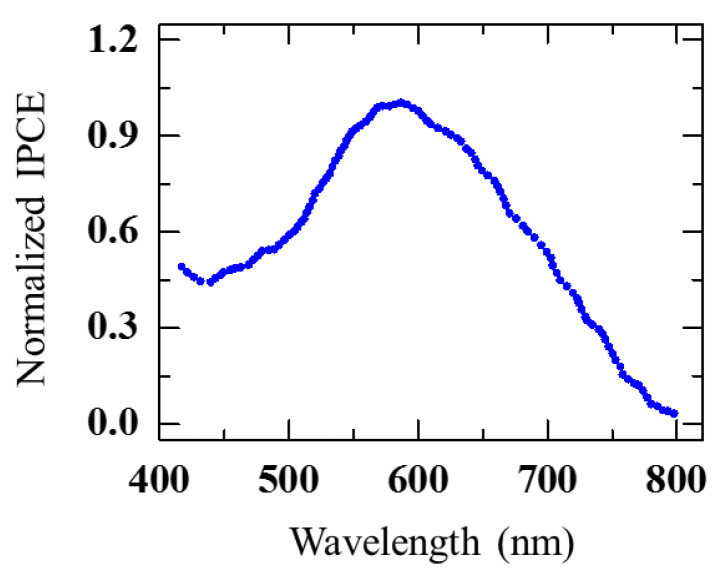
Measured IPCE (normalized) vs. incident wavelength (nm). Adapted from [86] with permission from the PCCP Owner Societies, 2017.

The plasmon losses over a wide range of energies (0.6–2.4 eV) underline the participation of Au in absorbing light over a wide range of energies (vis-near IR). To correlate the plasmonic effects with the device photoresponse, the device was irradiated with Xenon arc lamp source to capture the normalized incident photon-to-current conversion efficiency (IPCE) (Figure 14). 

The recorded IPCE, plotted against the impinging light at different wavelengths, increased with increasing wavelength, reaching the highest IPCE value for 590 nm, corresponding to 2.1 eV photon energy. In comparison with the EELS measurements, one can clearly notice that the contribution from the FH-like structure matches with this range (Figure 13g). Hence, the LSPR modes originating from the FH-like structure exhibit a very strong absorption at around 2.0–2.3 eV, close to the peak value obtained from the measured IPCE. This indicates that the WS device shows high LSPR activity when the energy of the incident photons is near to 2.1 eV (Figure 13g). In addition to the FH particles, there was a large number of smaller particles inside the cavities of the WS nanostructures [86]. These particles also contribute to the device’s photoresponse when immersed in a water solution to form a complete circuit.

The contribution from the interband transitions occurring in the Au particles can also be visualized in the IPCE measurement. At smaller wavelengths of less than 520 nm, the IPCE values are expected to be mostly generated by interband transitions. As in Au, the d bands lie at 2.4 eV (517 nm), just below the Fermi level; consequently, surface plasmons decay and hot electrons generation at 2.4 eV onwards (below 517 nm) are mostly expected via the interband transition.

As a summary, three cases studies corresponding to different strategies were demonstrated to highlight the role of material engineering in terms of design, morphology, size, and shape to enhance photocatalytic activity of Au/TiO_2_ systems by means of plasmonic effects. In conjunction with light absorption and photo-induced current measurements, various multiscale tools such as SNOM and HRSTEM-EELS were utilized to capture the hot spots that are the signature of LSPR. These hot spots are correlated to plasmonic effects on WS device activity. The active role of plasmonic nanoparticles in generating hot electrons, to be injected into the semiconductor conduction band, was showcased and explained by the light trapping mechanism and the shape of nanostructures. Note that it is highly important to bring the photocatalytic WS in the presence of plasmonic nanostructures a step forward to be comparable to high cost proven electrochemical WS systems. 

Certainly, photocatalytic WS for hydrogen production is a facile and viable process at very low operating costs, thanks to the unlimited resources such as water and sunlight. Nevertheless, it still faces real challenges to be scaled up due to the low overall efficiency. Many possibilities are in progress to cope with these challenges. For instance, the use of heterostructures made of hybridization of two types of semiconductors such as low band gap p-type semiconductor and high band gap n-type semiconductor where the energy band offset at the heterojunction interface can yield an efficient charge separation [87]. Similarly, two low-band gap semiconductors are mixed where the first one is intended to enhance the oxidation reaction and the second one with negative conduction band can promote the reduction reaction [87,88]. This mechanism, known as Z-scheme, can be used with or without electrons carrier [89,90]. For instance, carbon-based nanostructures such as two-dimensional graphene and gC_3_N_4_, were reported as electrons carriers due to their excellent electronic properties [91]. Other two-dimensional semiconductors, in particular metal dichalcogenides such as MoS_2_, were also reported to provide with high surface area while absorbing light in visible region [92,93]. Recently, an attempt to use hybrid systems such as photoelectrochemical solar WS based on MoS_2_ and III–V materials as photoelectrodes showed high WS performances. This could pave the way to a new generation of photo-electrochemical systems with lower power consumption for hydrogen production [94].

## 4. Conclusions

The contribution of plasmonic/photocatalyst nanostructures to efficient WS reaction and HER is ensured by two key properties: (1) as metal–semiconductor junction, they play the role of hole-scavengers and electron donors; and (2) as LSPR sites, they enhance the UV/vis absorption and reduce electron–hole diffusion length. Particularly, in this review, the contribution of Au/TiO_2_ plasmonic nanostructures is thoroughly discussed and investigated using advanced analytical techniques such as HRTEM-EELS and SNOM in conjunction with optical characterizations and photo-induced current measurements. The generated plasmonic hot spots observed with microscopy were reconstructed and validated using FDTD simulations. Our results are consistent with the fact that the LSPR material (Au) has extended the photocatalyst (TiO_2_) from UV to full light spectrum leading to the generation of photo-induced electrons which play an important role to achieving efficient water splitting. Nevertheless, additional investigations are still required to quantify accurately the WS efficiency and make proper connections among material selection, material design and quantification of produced hydrogen. This step is important before further development towards mass production of efficient WS device.
